# A Single Central Pattern Generator for the Control of a Locomotor Rolling Wave in Mollusc *Aplysia*

**DOI:** 10.34133/research.0060

**Published:** 2023-03-06

**Authors:** Hui-Ying Wang, Ke Yu, Zhe Yang, Guo Zhang, Shi-Qi Guo, Tao Wang, Dan-Dan Liu, Ruo-Nan Jia, Yu-Tong Zheng, Yan-Nan Su, Yi Lou, Klaudiusz R. Weiss, Hai-Bo Zhou, Feng Liu, Elizabeth C. Cropper, Quan Yu, Jian Jing

**Affiliations:** ^1^ State Key Laboratory of Pharmaceutical Biotechnology, Institute for Brain Sciences, Chinese Academy of Medical Sciences Research Unit of Extracellular RNA, Jiangsu Engineering Research Center for MicroRNA Biology and Biotechnology, Advanced Institute for Life Sciences, Chemistry and Biomedicine Innovation Center, School of Life Sciences, Nanjing University, Nanjing, Jiangsu 210023, China.; ^2^ National Laboratory of Solid State Microstructures, Department of Physics, Institute for Brain Sciences, and Collaborative Innovation Center of Advanced Microstructures, Nanjing University, Nanjing, Jiangsu 210093, China.; ^3^ Department of Neuroscience and Friedman Brain Institute, Icahn School of Medicine at Mount Sinai, New York, NY 10029, USA.; ^4^ School of Electronic Science and Engineering, Nanjing University, Nanjing, Jiangsu 210023, China.; ^5^ Peng Cheng Laboratory, Shenzhen 518000, China.

## Abstract

Locomotion in mollusc *Aplysia* is implemented by a pedal rolling wave, a type of axial locomotion. Well-studied examples of axial locomotion (pedal waves in *Drosophila* larvae and body waves in leech, lamprey, and fish) are generated in a segmented nervous system via activation of multiple coupled central pattern generators (CPGs). Pedal waves in molluscs, however, are generated by a single pedal ganglion, and it is unknown whether there are single or multiple CPGs that generate rhythmic activity and phase shifts between different body parts. During locomotion in intact *Aplysia*, bursting activity in the parapedal commissural nerve (PPCN) was found to occur during tail contraction. A cluster of 20 to 30 P1 root neurons (P1Ns) on the ventral surface of the pedal ganglion, active during the pedal wave, were identified. Computational cluster analysis revealed that there are 2 phases to the motor program: phase I (centered around 168°) and phase II (centered around 357°). PPCN activity occurs during phase II. The majority of P1Ns are motoneurons. Coactive P1Ns tend to be electrically coupled. Two classes of pedal interneurons (PIs) were characterized. Class 1 (PI1 and PI2) is active during phase I. Their axons make a loop within the pedal ganglion and contribute to locomotor pattern generation. They are electrically coupled to P1Ns that fire during phase I. Class 2 (PI3) is active during phase II and innervates the contralateral pedal ganglion. PI3 may contribute to bilateral coordination. Overall, our findings support the idea that *Aplysia* pedal waves are generated by a single CPG.

## Introduction

Locomotion is arguably one of the most fundamentally important behaviors an animal possesses, as it enables the animal to explore the environment, to approach prey, and to avoid predation, all essential for its survival and fitness [1–3]. Thus, it is critical to understand neural mechanisms underlying locomotion. Based on how the propulsive force is generated, locomotion can be classified into 2 major forms: appendicular locomotion, which depends on appendage movement (e.g., vertebrate limb locomotion and bird and insect wing flight), and axial locomotion, which depends on body or pedal movement. One of the most common forms of axial locomotion is the rolling wave. Examples include pedal waves in *Drosophila* larvae and *Aplysia*, and body waves in *Caenorhabditis elegans*, leech, lamprey, and fish [4–11]. Although rolling waves are present in diverse species and may differ in how they are implemented, they share certain characteristics. All involve rhythmic contractions of body segments or the foot and require phase shifts between areas undergoing contraction. Currently, neural mechanisms underlying axial locomotion in *C. elegans*, *Drosophila* larvae, leech, lamprey, and zebrafish have been studied and characterized to various degrees [5–15]. These studies showed that most rolling-wave locomotor behaviors are mediated by multiple segmental CPGs that are coupled.

In contrast to other species, *Aplysia*, like other molluscs, does not have a segmented central nervous system (CNS) and its locomotion is mediated by a single ganglion, the pedal ganglion. In principle, axial movements in *Aplysia* could result from the activity of multiple coupled CPGs within the pedal ganglion. Alternatively, a mechanism for producing phase shifts could be included in a single CPG. In this study, we sought to address this question by studying the motor organization and pattern-generating mechanisms for the pedal wave in *Aplysia*.

The *Aplysia* pedal wave is characterized by rhythmic contractions of the foot propagating from the front to the back [4]. Previous work on *Aplysia* locomotion has provided information on pedal ganglion neurons involved in motor control [4,16–18] and has identified higher-order neurons in the cerebral ganglion that may initiate a defensive form of the behavior, e.g., CC9/10 [19,20]. Existing data also suggest that a putative pattern-generating network is located in the pedal ganglion [17,21], but pattern-generating mechanisms have not been elucidated. Thus, an open question of fundamental importance is, how is rhythmic activity and the phase shift that creates the pedal wave generated?

In *Aplysia*, the parapedal commissural nerve (PPCN) (also referred to as P10), which innervates the foot, has been used as a monitor for locomotion [16–20]. Although PPCN/P10 activity has been recorded in intact animals [22], it is not clear which part of the foot is actually contracting when this activity occurs. In addition, despite extensive recordings of locomotor activity from neurons on the dorsal surface of the pedal ganglion [4,16–18], it remains unclear how many of these dorsal neurons are motoneurons. More importantly, pedal interneurons (PI) have not yet been identified.

Here, we demonstrate that PPCN activity is recorded during the contraction of the tail/posterior foot in intact animals. On the basis of this activity pattern, we describe a newly identified cluster of neurons on the ventral surface of the pedal ganglion, which we refer to as P1 root neurons (P1Ns). We also describe 3 PIs on the ventral surface of the ganglion that contribute to rhythm generation and the generation of the phase shift. Cluster analysis of phases of the P1N population showed that there were 2 clusters in locomotor programs, and the 2 cluster centers matched phasing of the 2 classes of interneurons. Finally, we demonstrate that there is extensive electrical coupling among P1Ns, and between P1Ns and coactive interneurons. These and additional data allowed us to propose a pattern-generating network based on a half-center oscillator, which is responsible for a rolling wave in an animal with a CNS that is not segmented.

## Results

### The PPCN/P10 as a monitor for locomotion

When *Aplysia* locomotes, a rolling wave is generated, i.e., muscle contractions progress from the front to the back of the foot (i.e., the tail) during each cycle. Our initial experiments sought to identify a peripheral nerve that could be used to monitor an identified phase of this rolling wave. Previously, the PPCN, also named P10, has been used for this purpose [16–20,23,24]. Bursting activity has been recorded from P10/PPCN during nerve-induced locomotor activity in semi-intact preparations [16] and in intact animals [22]. Although PPCN innervates the posterior foot [16], based on visual observations, an early report [22] stated that bursts of P10/PPCN activity occur during the neck shortening phase of a locomotor step, i.e., when the anterior foot contracts. This study did not, however, include quantitative data to support this claim.

**Fig. 1.**
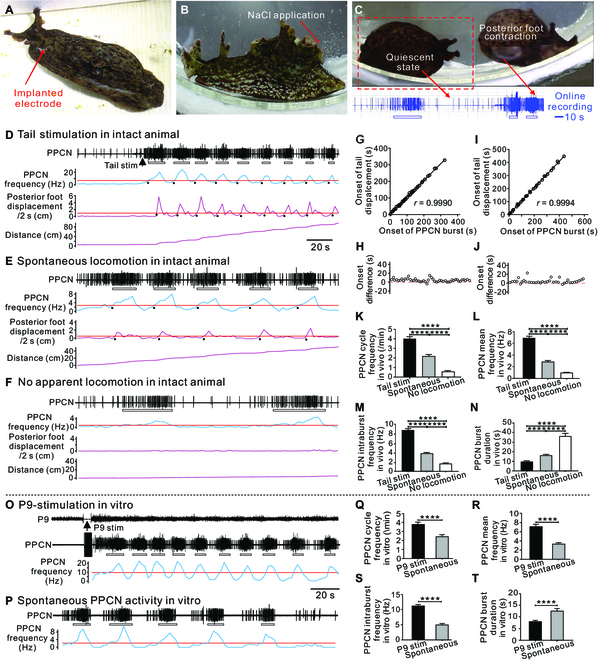
PPCN activity during locomotion in intact animals and during locomotor programs in the isolated CNS. (A) Photograph showing an intact animal with an implanted electrode. (B) Photograph showing NaCl application to the tail of an animal. (C) PPCN recording (below) during locomotor behavior in an intact animal. Electrical activity was not observed when the animal was in a quiescent state, but was observed during locomotion when the posterior foot contracted. (D to F) PPCN and movement data from intact animals during NaCl-triggered locomotion (D), during spontaneous locomotion (E), and in the situation where there was no apparent locomotion (F). In all 3 cases, the top plot (blue) is the combined firing frequency of all units detected in the PPCN, and the other 2 plots (purple) were obtained from analysis of video records. The middle plot indicates the speed of tail/posterior foot movement (displacement/time is plotted), and the bottom plot indicates the total distance traveled. (G to J) Quantification of onset of PPCN bursts and posterior foot displacement, defined as the time when the frequency of a PPCN burst or the posterior foot displacement crosses the average value (red lines), which is illustrated as a black dot in (D) and (E). (G and H) Locomotion after NaCl application to the tail (*n* = 47 from 6 preparations). (I and J) Spontaneous locomotion (*n* = 36 from 6 preparations). (G and I) Correlation analysis. (H and J) Onset difference: onset of posterior foot displacement − onset of a PPCN burst. (K to N) Group data for PPCN activity in intact animals during NaCl-triggered and spontaneous locomotion and in the situation where there was no apparent locomotion (one-way ANOVA: (K) is a plot of the PPCN cycle frequency, *F*(2,57) = 85.74, *P* < 0.0001; (L) is a plot of mean frequency, *F*(2,57) = 156.3, *P* < 0.0001; (M) is a plot of intraburst frequency, *F*(2,57) = 168.1, *P* < 0.0001; (N) is a plot of burst duration, *F*(2,57) = 53.10, *P* < 0.0001. Bonferroni post hoc test, ****P < 0.0001, *n* = 20. (O and P) PPCN activity after P9 stimulation (P9 stim) (O) and during spontaneous locomotor programs (P) in the isolated CNS. (Q to T) Paired *t* tests comparing cycle frequency (Q), mean frequency (R), intraburst frequency (S), and burst duration (T) (****P < 0.0001, *n* = 21). PPCN bursts are defined based on frequency plots (blue traces) with binned data (3-s bins). Red horizontal lines indicate the average PPCN firing frequency or posterior foot displacement over the entire recordings illustrated and are used to mark the beginning and the end of PPCN bursts/posterior foot movement. Open bars below PPCN traces mark bursting activity. Error bars, SEM.

To resolve this apparent controversy, we recorded PPCN activity in intact animals using an implanted electrode (Fig. 1A) and found that there was bursting activity during defensive locomotion elicited by NaCl application to the tail (Fig. 1B to D), and in contrast to the previous report [22], bursts of activity preceded and overlapped with the contraction of the tail or the posterior foot. This was evident when video recordings were viewed (Fig. 1C and Movie S1), and confirmed when the posterior foot displacement data were quantified (Fig. 1D and Fig. S1A). For example, we found that PPCN burst onset was positively correlated with the onset of the posterior foot contraction (Fig. 1G), and that the former preceded the latter (Fig. 1H) (*n* = 47 from 6 preparations). In contrast, PPCN bursts were out of phase with contractions of the front foot (Fig. S1B). Similar findings were obtained when we analyzed data obtained during spontaneous locomotion (Fig. 1E, I, and J and Fig. S1C; *n* = 36 from 6 preparations). Overall, we conclude that PPCN/P10 bursting activity is associated with the tail/posterior foot contraction, rather than the contraction of the front foot or the neck.

Studies of the neural basis of locomotion are often conducted in in vitro preparations in which activity is triggered via nerve stimulation. To be able to compare activity induced in this manner to activity recorded in intact animals, we further characterized in vivo activity. We compared PPCN bursts observed during defensive locomotion elicited by NaCl to bursts that occurred spontaneously in the situation where locomotion was observed, and in the situation where it was not (Fig. 1D to F and K to N). The PPCN cycle frequency was highest during defensive locomotion (Fig. 1K), as was the mean firing frequency of PPCN units (Fig. 1L), and the intraburst frequency (Fig. 1M) (see “Data and statistical analyses” section in Materials and Methods). The burst duration was shorter (Fig. 1N). When the intraburst frequency was about 2 Hz or lower, locomotor movements were not observed (Fig. 1M). These data indicate that the mean and intraburst firing frequencies are the highest with locomotion evoked by NaCl, and are the lowest when there is no movement.

In vitro, we triggered defensive locomotor programs using the tail nerve (i.e., P9). Again, defensive activity differed from spontaneous activity (Fig. 1O to T). Namely, the PPCN cycle frequency (Fig. 1Q), mean frequency (Fig. 1R), and intraburst frequency (Fig. 1S) were higher during defensive locomotion, while the burst duration was shorter (Fig. 1T). Notably, in vitro activity triggered by P9 stimulation was similar to activity triggered by NaCl in intact animals (e.g., the cycle frequency for defensive locomotion was 3.97 ± 0.25 Hz in vivo and 3.82 ± 0.23 Hz in vitro, and PPCN mean frequency was 6.91 ± 0.34 Hz in vivo and 7.23 ± 0.46 Hz in vitro).

To identify neurons with processes in the PPCN, we backfilled PPCN using biocytin that was visualized with a fluorescence dye. We observed labeling in 3 to 4 medium-sized somata on the dorsal surface of the pedal ganglion (*n* = 3). The 3 neurons clearly had axons in the PPCN, and their medium- to large-sized cell bodies support that they are putative motoneurons (Fig. S2). In fact, we observed units with different amplitudes in PPCN recordings that could represent the different backfilled neurons (see Fig. 1D, O, and P).

Thus, we provide the first direct evidence that PPCN bursts occur before and during the tail contraction of both defensive and spontaneously occurring locomotion in intact animals, and PPCN activity is similar in in vivo and in vitro preparations.

### Motor organization for locomotion

PPCN activity is confined to a single phase of the locomotor program. We therefore conducted further experiments to identify putative motoneurons that could represent the entire cycle of the rolling wave. To accomplish this, we initially focused on the dorsal surface of the pedal ganglion since previous studies have demonstrated that this surface contains neurons that burst during fictive locomotion in semi-intact preparations and in the isolated CNS [4,17,18].

**Fig. 2.**
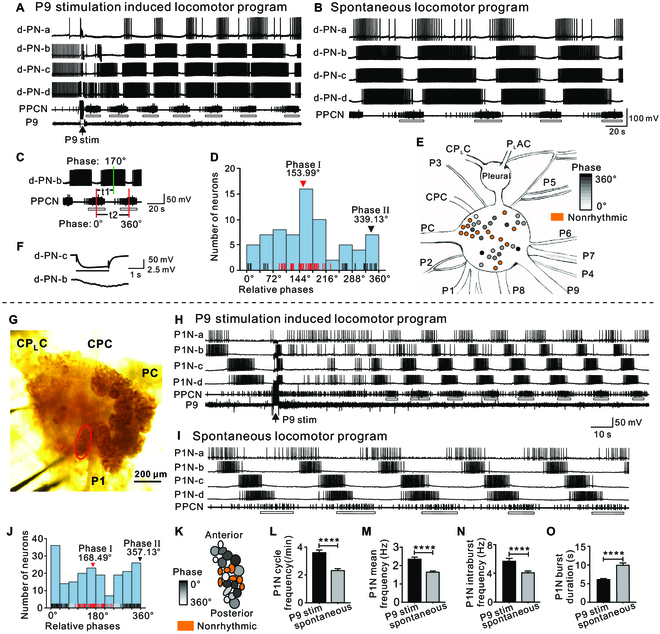
Rhythmically active neurons in the pedal ganglion. (A to E) Neurons on the dorsal surface of the pedal ganglion. (A and B) Activity of 4 dorsal pedal neurons (d-PNs) and the PPCN during P9-induced (A) and spontaneous locomotor programs (B). Arrow: P9 stimulation (P9 stim). (C) Definition of phase relationships relative to PPCN bursts. In all cases, the center of the burst was used to define the phase of activity. t2: the duration of a full cycle of PPCN bursting activity (2 red vertical lines: 0°, 360°). t1: the elapsed time from the bursting of d-PN-b (green vertical line) relative to the PPCN bursting (the first red vertical line). The phase of the d-PN-b is defined as “t1/t2*360° (range from 0° to 360°), which is 170°. (D) Histogram showing the phase distribution of 71 rhythmically active d-PNs recorded in 4 preparations. Each data point (phase of a dorsal pedal neuron) is shown as a short red, gray, or black vertical line along the *x* axis. Red lines belong to the phase I (153.99°) cluster, black lines belong to the phase II (339.13°) cluster, whereas gray lines are data points that could not be assigned stably to one cluster during 10 independent *K*-means clustering trials. (E) Locations of cell bodies of rhythmically active d-PNs in the pedal ganglion of a single preparation. Activity phasing is indicated by the shading. Nonrhythmic neurons are colored orange. CPC, cerebropedal connective; CP_L_C, cerebropleural connective; P1 to P9, pedal nerve 1 to 9; PC, pedal commissure; P_L_AC, pleuroabdominal connective; Pleural, pleural ganglion. (F) Representative example showing electrical coupling between 2 d-PNs that were coactive (the phase of d-PN-b is 170.3° ± 2.6°, and that of d-PN-c is 178° ± 1.4°). (G to O) Neurons on the ventral surface of the pedal ganglion. (G) Photograph showing a cluster of P1Ns (indicated by the red oval). CPC, cerebropedal connective; CP_L_C, cerebropleural connective; P1, pedal nerve 1; PC, pedal commissure. (H and I) Representative examples of rhythmic activity of P1Ns and activity in the PPCN during P9-elicited (H) and spontaneous (I) locomotor programs. Note the various phases of individual P1Ns relative to PPCN. Arrow: P9 stimulation (P9 stim). (J) Phase distribution of 200 rhythmically firing P1Ns in 19 preparations. Each data point (phase of a P1N) is shown as a short red, gray, or black vertical line along the *x* axis. Red lines belong to phase I (168.49°) cluster, black lines belong to phase II (357.13°) cluster, whereas gray lines are data points that could not be assigned stably to one cluster during 10 independent *K*-means clustering. (K) Schematic diagram illustrating the locations of cell bodies in the P1 root cluster. Activity phasing indicated by different grayscales. Neurons not rhythmically active are shown in orange. (L to O) Comparison of P1N cycle frequency (L), P1N mean frequency (M), P1N intraburst frequency (N), and P1N burst duration (O) (paired *t* test, ****P < 0.0001). Error bars, SEM. Open bars below PPCN traces mark bursting activity.

To confirm these results and characterize the relative phasing between rhythmically active neurons, we recorded from them during spontaneous motor programs (Fig. 2B), and motor programs triggered by P9 (Fig. 2A). To quantify relative phasing, we used bursts of activity in the PPCN as a frame of reference (Fig. 2C). Namely, we identified the midpoint of each burst of activity and determined its position relative to the midpoint of the PPCN burst that immediately preceded it. If the 2 midpoints overlapped, the phase was defined as 0° or 360° (i.e., activity was in phase). Activity was out of phase at 180° when the burst occurred at the midpoint between 2 adjacent PPCN bursts, e.g., d-PN-b fired at 170° (Fig. 2C). For 71 dorsal neurons in 4 preparations, relative phasing was distributed between 0° and 360° (Fig. 2D). This confirms the previous suggestion that these cells could, in principle, represent an entire cycle of the rolling wave (see also [18]).

However, visual inspection of the data (Fig. 2D) suggested that phasing is not uniformly distributed. A Pycke test supported this (*P* = 0.006; Table and Fig. 2D). Data were then analyzed by running Rayleigh tests (Table). An initial analysis detected a single peak of activity at 141.5°. However, a 2-fold transformation of the data suggested an alternative possibility, i.e., 2 symmetrical cluster peaks at 159.8° and 339.8°. We also tested for 3 peaks by performing a 3-fold data transformation, but these results were negative. If there are 2 peaks, it is possible that we failed to detect the second peak with the initial analysis because the cluster around 141.5° to 159.8° (subsequently named phase I) was more prominent than the cluster around 339.8° (subsequently named phase II). To explore this possibility, we further analyzed data using a different method: *K*-means clustering (Fig. 2D; for more extensive analyses, see the Supplementary Materials). Neurons that were consistently assigned in the first or second cluster on 10 independent clustering trials are illustrated with red or black vertical lines, whereas neurons that were assigned to either cluster are indicated with gray vertical lines. *φ*_k,center_ for the 2 clusters is indicated at the top of the histogram. We refer to the cluster that has *φ*_k,center_ of 153.99° as phase I, and that of 339.13° as phase II. Note that these 2 cluster peaks were similar to those calculated using Rayleigh vector (Table). Taken together, these data indicate that although there are rhythmically active locomotor neurons on the dorsal surface of the pedal ganglion that are active throughout motor programs, there are 2 distinct peaks of activity. We also mapped the positions of these rhythmically active neurons and found that they were distributed over the entire dorsal surface and were intermingled with nonbursting neurons (Fig. 2E). Consistently, the PPCN backfill showed that dorsal neurons innervating PPCN (i.e., the 2 neurons on the left pedal ganglion in Fig. S2A) appear to be also distributed, not clustered.

**Table.** Results for statistical and cluster analyses for circular data of the phases of pedal neurons

Data		Pycke test	Rayleigh test			*K*-means clustering
			Original data	2-fold transformation	3-fold transformation	
Dorsal neurons (Fig. 2D, *n* = 71 cells)	*P* value	0.006, nonuniform	0.02, one cluster	0.02, 2 clusters	0.71, no	N/A
	Cluster centers		141.5°	159.8°, 339.8°	N/A	153.9°, 339.1°
P1Ns (Fig. 2J, *n* = 200 cells)	*P* value	0.008, nonuniform	>0.05, no	0.007, 2 clusters	0.52, no	N/A
	Cluster centers		N/A	175.7°, 355.7°	N/A	168.5°, 357.1°
Coupled P1Ns (Fig. 4B, *n* = 113 cells)	*P* value	0.005, nonuniform	>0.05, no	5.7 × 10^−5^, 2 clusters	0.34, no	N/A
	Cluster centers		N/A	155.7°, 335.7°	N/A	151.6°, 330.9°
Functions of a test/analysis	Test for nonuniformity	Test for one cluster	Test for 2 symmetric clusters	Test for 3 symmetric clusters	Cluster analysis

Previous studies have not described locomotor neurons on the ventral surface of the pedal ganglion; we therefore performed an additional set of experiments to explore this region. Again, we recorded from neurons during spontaneous motor programs and programs triggered by P9. We observed bursting activity in neurons that were part of a cluster of 20 to 30 medium-sized cells near the root of the P1 nerve. We refer to these P1 root neurons as P1Ns (Fig. 2G). Sixty-seven percent of the P1Ns we recorded showed bursting activity during the time that PPCN bursting activity was recorded (Fig. 2H and I). Using the analyses method described above (Fig. 2C to E), we found that ventral cells were similar to dorsal cells in that activity was observed throughout motor programs. Namely, for 200 rhythmically firing P1Ns in 19 preparations, phases ranged from 0° to 360° (Fig. 2J). Again, activity was not uniformly distributed (Fig. 2J; Pycke test, *P* = 0.007). Rayleigh tests were run on both original and transformed data (Table), showing that there were 2 symmetrical cluster peaks, in this case at 175.7° and 355.7°. *K*-means clustering (Fig. 2J, see also the Supplementary Materials) determined that *φ*_k,center_ equaled 168.49° and 357.13° for the first and second phase, respectively, similar to cluster peaks as determined by Rayleigh vector (Table). These values are similar to those obtained in experiments that characterized the dorsal pedal neurons (Fig. 2D). Interestingly, there are more phase I neurons on the dorsal surface and more phase II neurons on the ventral surface. The distribution of the ventral P1Ns is shown in Fig. 2K, with phase indicated by the gray shading. Neurons that were coactive were not necessarily adjacent to each other.

We computed the average cycle frequency (Fig. 2L), mean frequency (Fig. 2M), intraburst frequency (Fig. 2N), and burst duration (Fig. 2O) of the activity of 47 identified P1Ns in 23 preparations. As with PPCN activity (Fig. 1K to N), the 3 former parameters were higher when evoked activity was compared to spontaneous activity (Fig. 2L to N), whereas the burst duration was shorter (Fig. 2O).

**Fig. 3.**
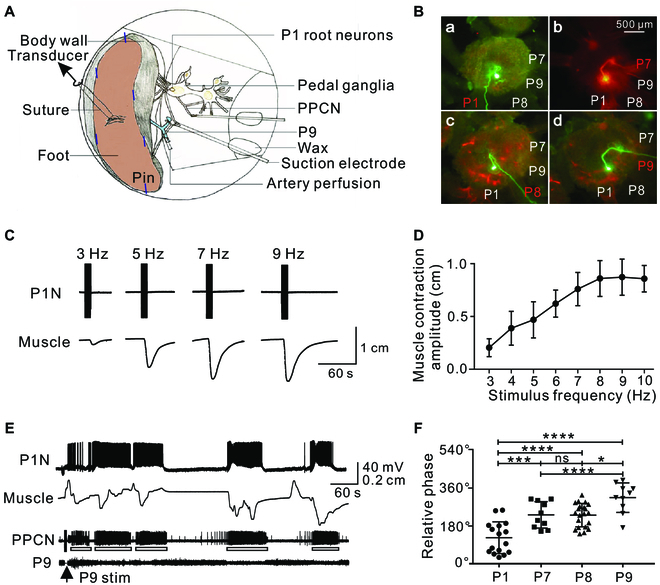
Motoneuron–muscle relationship. (A) Schematic diagram of the neuromuscular setup. Intracellular recordings were obtained from P1Ns using glass microelectrodes. Extracellular recordings from P9 were made en passant. PPCN recordings were made using suction electrodes. Arteries are shown in light blue. (B) Morphology of P1Ns that had axons projecting to P1 (a, *n* = 16), P7 (b, *n* = 10), P8 (c, *n* = 21), and P9 (d, *n* = 10) injected with Alexa 488 (green) or 568 (red) dyes. P1-P9: pedal nerve 1 to 9. (C) Representative example showing that stimulation of a P1N for 10 s at increasing frequencies induced progressively larger foot muscle contractions. (D) Group data (*n* = 7, one-way ANOVA, *F*(7,32) = 2.867, *P* = 0.0193). (E) After P9 stimulation (arrow), the phasic activity of a P1N was associated with rhythmic contractions of the foot muscle and PPCN bursting activity (*n* = 7). (F) Plot of phases of P1Ns versus their projections via different peripheral nerves, P1, P7, P8, and P9. One-way ANOVA: *F*(3,53) = 18.73, *P* < 0.0001; Bonferroni post hoc test, ns, *P* > 0.05; *P < 0.05; ***P < 0.001; ****P < 0.0001. Error bars, SEM. Open bars below PPCN traces mark bursting activity.

To further characterize rhythmically active neurons and determine whether they are motor neurons, we took advantage of the fact that these ventral neurons are part of a recognizable cluster. To determine whether these cells are likely to be motoneurons, we first injected P1Ns with fluorescent dyes. All neurons that were injected had axons in peripheral pedal nerves, such as P1, P7/8, and P9 (Fig. 3B). Second, to determine whether there is a motor innervation, we intracellularly stimulated P1Ns and recorded changes in muscle length in semi-intact preparations (Fig. 3A). We found that muscle contractions were elicited by 59% of the P1Ns tested (i.e., 100 of 169 neurons in 55 animals) and that the magnitude of muscle contractions increased in a frequency-dependent manner (Fig. 3C and D). The frequency that could elicit a muscle contraction ranged from 3 to 9 Hz (Fig. 3D), which matched the frequency range recorded during locomotor programs (Fig. 2N). This indicates that the majority of the P1Ns are motoneurons.

When locomotor activity was induced by stimulating P9 in semi-intact preparations, P1N activity was correlated with contraction of foot muscles (*n* = 7; Fig. 3E). The pedal nerves innervate different parts of the foot, with P1 innervating the anterior part, P7/8 the middle part, and P9 the posterior part [16,25]. As expected, neurons with projections in P1 tended to be active during the early phase of locomotion (124.4°, close to phase I), those projecting to P7/8 tended to be active during the middle phase (231°), and those projecting to P9 tended to be active during the late phase (313°, phase II) (Fig. 3F).

### Synaptic connections between pedal neurons

Previous studies using intracellular recording techniques did not find synaptic connections between dorsal neurons [4,17]. Here, we tested a large number of dorsal cells (223 pairs of neurons in 13 animals), and although electrical coupling was relatively rare, we did identify 5 pairs of coupled neurons (Fig. 2F). We determined the coupling ratio for these neurons. The average coupling ratio was 0.0146 ± 0.0062. Neurons that were electrically coupled fired during the same phase of the motor program (e.g., in Fig. 2F, the phase of d-PN-b was 170.3° ± 2.6°, and that of d-PN-c was 178° ± 1.4°).

**Fig. 4.**
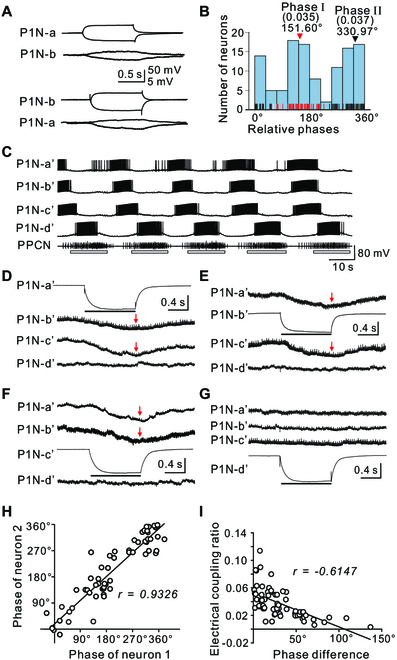
Electrical coupling between P1 root neurons. (A) Electrical coupling between 2 P1Ns that fired during phase I of the motor program as shown in Fig. 2 (H and I) (phase of P1Ns: P1N-a: 136.2° ± 2.1°, P1N-b: 164.4° ± 3.2°). Coupling was measured by injecting both depolarizing and hyperpolarizing pulses. The coupling ratio was similar in the 2 situations, indicating that the electrical coupling is nonrectifying. (B) Histogram of electrically coupled P1Ns active during different phases. Each data point (phase of an electrically coupled P1N) is shown as a short red, gray, or black vertical line along the *x* axis. Red lines belong to phase I (151.6°, average coupling ratio: 0.035 ± 0.0028) cluster, black lines belong to phase II (330.97°, average coupling ratio: 0.037 ± 0.0026) cluster, whereas gray lines are data points that could not be assigned stably to one cluster during 10 independent *K*-means clustering trials. The data show that P1Ns near phases I and II were more likely to be coupled. (C) Motor program elicited by P9 stimulation showing simultaneous activity of 4 rhythmically active P1Ns (P1N-a′ to P1N-d′). P1N-a′, P1N-b′, and P1N-c′ showed similar phasing, whereas P1N-d′ was active when PPCN activity was recorded (phases: P1N-a′: 186.3° ± 2.7°; P1N-b′: 197.1° ± 1.6°; P1N-c′: 205.2° ± 1.4°; P1N-d′: 334.3° ± 1.6°). (D to G) Potential electrical coupling between the 4 cells shown in (C). Injection of a negative current pulse in P1N-a′ (D), P1N-b′ (E), or P1N-c′ (F) hyperpolarized the other 2 neurons, but did not change the membrane potential of P1N-d′. Injection of a negative current pulse in P1N-d′ (G) did not change the membrane potential of P1N-a′, b′, or c′. Thus, P1N-a′, P1N-b′, and P1N-c′ were all electrically coupled to each other, but not to P1N-d′. Vertical calibration: 50 mV for the neuron that received the current injection, and 2 mV for the other neurons. Red arrows indicate the time when the current injection was terminated. (H) Plot of phases of coupled neuron pairs, showing that the coupled pairs tended to have similar phases (*n* = 65 pairs). (I) Plot of the electrical coupling ratio versus phase difference of the coupled pairs showing that the coupled pairs with smaller phase differences tended to have higher coupling ratios (*n* = 65 pairs). r. Pearson r. Open bars below PPCN traces mark bursting activity.

In contrast, we found extensive electrical coupling among ventral P1 root neurons, i.e., ~43% (113 of 262 P1Ns tested) were coupled (Fig. 4A and D to G). Electrical coupling appeared to be nonrectifying because coupling ratios measured by hyperpolarizing or depolarizing pulses were similar (Fig. 4A). Coupling was particularly common between neurons that were active during a similar phase of the motor program (e.g., in Fig. 4A: P1N-a: 136.2° ± 2.1°, P1N-b: 164.4° ± 3.2°; Fig. 4C to G, P1N-a′: 186.3° ± 2.7°; P1N-b′: 197.1° ± 1.6°; P1N-c′: 205.2° ± 1.4°; P1N-d′: 334.3° ± 1.6°). That is, coupling was observed between 82 and 113 coactive neurons in 27 preparations, whereas coupling was only observed between 31 and 113 neurons that fired during different phases of the motor program (Fig. 4B and H). The smaller the phase difference between 2 coupled neurons, the higher electrical coupling ratios became (Fig. 4I). A Pycke test revealed a nonuniform distribution of phases among 113 coupled P1Ns in 27 preparations (*P* = 0.002, Fig. 4B). Rayleigh tests (Table) demonstrated that there were 2 symmetrical cluster peaks at 155.7° and 335.7°. *K*-means clustering (Fig. 4B, see also the Supplementary Materials) showed that *φ*_k,center_ was around 151.6° and 330.97° for the first and second cluster, respectively, similar to cluster peaks as determined by Rayleigh vector (Table). These data indicate that electrical coupling plays a role in synchronizing the activity of coactive P1Ns.

To summarize, we demonstrated that PPCN is a reliable monitor of locomotion because unit frequency is a good indicator of the type of locomotor behavior induced (Fig. 1). We also found a cluster of 20 to 30 neurons on the ventral surface of the pedal ganglion, which are rhythmically active during the pedal rolling wave. The activity of these neurons can be divided into 2 phases, i.e., phases I and II (Fig. 2G to O). A majority of these neurons are motoneurons (Fig. 3). Taken together, this activity of P1Ns combined with PPCN recordings is a good representation of the pedal rolling wave.

### Pattern-generating interneurons for locomotion

Previous investigators have extensively explored the dorsal surface of the pedal ganglion but failed to identify any locomotor interneurons [4,17]. We therefore focused on the ventral surface.

**Fig. 5.**
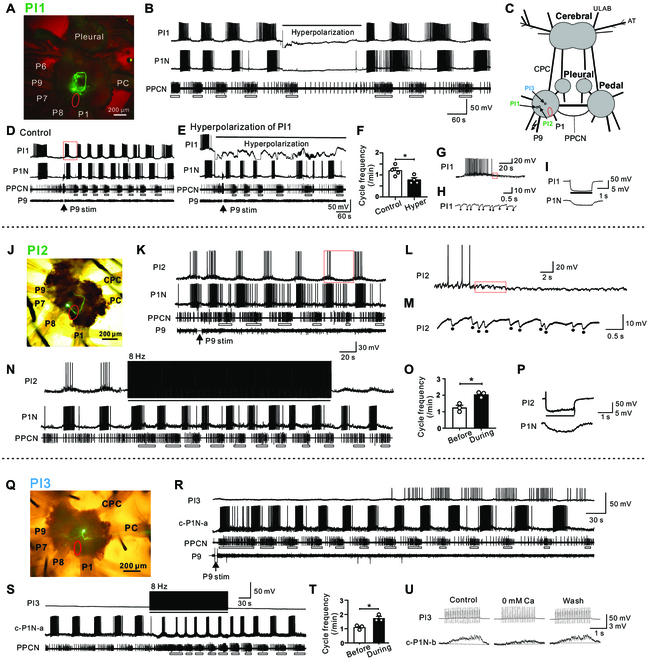
Characterization of pedal interneurons PI1, PI2, and PI3. (A to I) Properties of PI1. (A) Fluorescent image of PI1 (green). The red oval marks the location of P1Ns. (B) In a spontaneous locomotor program, PI1 fired rhythmically and a burst of activity was recorded every time there was a burst of activity in the PPCN. PI1 activity occurred at 157.1° ± 5.7°. Hyperpolarizing PI1 (bar) by injecting −4 nA current suppressed the occurrence of the locomotor program. (C) Schematic diagram showing the locations of the 3 interneurons and P1Ns (red oval) identified on the ventral surface. (D) After P9 stimulation (arrow), PI1, P1N (the activity phasing is 162.2° ± 6.2°), and PPCN were rhythmically active. (E and F) During hyperpolarization of PI1 with −4 nA current (bar), the cycle frequency was significantly reduced [(F) group data, paired *t* test, *P < 0.05, *n* = 4]. (G and H) PI1 received a large number of IPSPs from other neurons during the locomotor program induced by P9 stimulation. (G) is an enlarged view of the recording shown in the red square in (D), and (H) is an enlarged view of the recording shown in the red square in (G). The dots in (H) mark individual IPSPs. (I) Electrical coupling between PI1 and a P1N that fired during a similar phase of the motor program. Injecting a negative current into PI1 (bar) hyperpolarized P1N. (J to P) Properties of PI2. (J) Fluorescent image of PI2 (green). The red oval marks the location of P1Ns. (K) PI2 was rhythmically active during a locomotor program elicited by P9 stimulation (P9 stim, arrow). It fired at 164.4° ± 7.6°. (L and M) PI2 received a large number of IPSPs from other neurons during the locomotor program induced by P9. (L) is an enlarged view of the recording shown in the red square in (K), and (M) is an enlarged view of the recording shown in the red square in (L). The dots in (M) mark individual IPSPs. (N and O) Stimulating PI2 at 8 Hz (bar) enhanced the locomotor program [(O) group data, paired *t* test, *P < 0.05, *n* = 3]. (P) Electrical coupling between PI2 and a P1N that fired at a similar time. Injecting a negative current pulse into PI2 (bar) hyperpolarized P1N (the activity phasing is 167.4° ± 1.2°). (Q to U) Properties of PI3. (Q) Fluorescent image of PI3 (green) showing one branch of its axon projecting laterally, and the main axon projecting medially to the pedal commissure (PC), and eventually to the contralateral pedal ganglion. The red oval marks the location of the P1Ns. (R) PI3 was rhythmically active (the activity phasing is 342.0° ± 3.2°) during the latter part of a locomotor program elicited by P9 stimulation (P9 stim, arrow). c-P1N-a, contralateral P1N (the activity phasing is 125.08° ± 1.8°). (S and T) Stimulating PI3 at 8 Hz (bar) increased the cycle frequency of the locomotor program. [(T) group data, paired *t* test, *P < 0.05, *n* = 3]. (U) Stimulation of PI3 elicited fast EPSPs in the c-P1N-b (the activity phasing is 15° ± 3.39°) that followed presynaptic spikes one-for-one. Perfusion of preparations with 0 mM Ca^2+^ ASW did not completely block EPSPs, suggesting that they are partly chemical and partly electrical. Cerebral, cerebral ganglion; CPC, cerebral–pedal connective; AT, anterior tentacular nerve; P1, P9, pedal nerve 1 or 9; Pedal, pedal ganglion; PC, pedal commissure; PI, pedal interneuron; Pleural, pleural ganglion; PPCN, parapedal commissure nerve; ULAB, upper labial nerve. Error bars, SEM. Open bars below PPCN traces mark bursting activity.

**Fig. 6.**
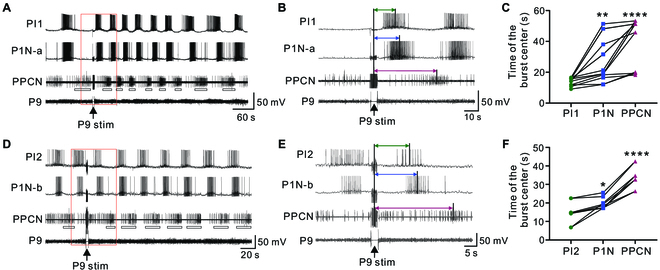
The timing of PI1/PI2 bursts of activity relative to bursts of P1N and PPCN activity immediately after P9 stimulation. (A to C) PI1. (A) Example showing rhythmic activity of PI1, P1N-a, and PPCN. (B) Expanded recording of the section of the record marked by a red box in (A). (C) Group data showing the timing of the burst centers for PI1, P1Ns, and PPCN [repeated-measures one-way ANOVA: *F*(2,26) = 33.25; *P* < 0.0001; Bonferroni post hoc test, PI1 versus P1N. **P < 0.001; and PI1 versus PPCN: ****P < 0.0001, *n* = 14 from 5 preparations]. (D to F) PI2. (D) Example showing rhythmic activity of PI2, P1N-b, and PPCN following P9 stimulation. (E) Expanded recording of the section marked by a red box in (D). (F) Group data showing the timing of the burst centers for PI2, P1Ns, and PPCN [repeated-measures one-way ANOVA: *F*(2,16) = 84.48; *P* < 0.0001; Bonferroni post hoc test, PI2 versus P1N: *P < 0.05; and PI2 versus PPCN: ****P < 0.0001, *n* = 9 from 3 preparations]. The data showed that PI1 and PI2 bursts tend to precede bursts of activity in P1N and PPCN.

We found 2 major classes of interneurons (Fig. 5C), which were initially identified based on their soma locations, soma size, and firing patterns during locomotor programs. At the end of physiological experiments, these interneurons were injected with carboxyfluorescein to reveal their axonal projections and therefore to confirm their identities. One class appeared to be active during phase I of the motor program, whereas the other class appeared to be active during phase II. We identified 2 members of the first class: PI1 (*n* = 4; Fig. 5A to I) and PI2 (*n* = 3; Fig. 5J to P). PI1 (soma size: ~38 μm) and PI2 (soma size: ~30 μm) had a similar morphology, i.e., their somata were located near the roots of P7 and P8, and their axons projected medially, anteriorly, and then medially, forming nearly a loop within the ipsilateral ganglion (Fig. 5A and J). Both neurons fired at ~160° (PI1: 157.1° ± 5.7°, *n* = 4; PI2: 164.4° ± 7.6°, *n* = 3) during locomotor programs elicited by P9 and during spontaneously occurring programs (Fig. 5B, D, K, and N). When they were inactive between bursting activity, both neurons received fast inhibitory postsynaptic potentials (IPSPs) (Fig. 5G, H, L, and M). Intriguingly, both neurons were electrically coupled to P1Ns that fired during a similar phase (162°-167°) with coupling ratios of 0.11 ± 0.014 (PI1, *n* = 3; Fig. 5I) and 0.12 ± 0.009 (PI2, *n* = 4; Fig. 5P). PI1 appeared to play a role in pattern generation in that, when it was hyperpolarized, spontaneous programs did not occur (Fig. 5E), and there was a reduction in cycle frequency when programs were triggered by P9 (Fig. 5D to F, *n* = 4). On the other hand, stimulation of PI2 promoted locomotor programs (Fig. 5N and O, *n* = 3). Thus, both PI1 and PI2 are part of the locomotor CPG. We also analyzed the timing of burst centers of PI1 and PI2 relative to that of burst centers of P1N and PPCN following P9 stimulation (Fig. 6). The data showed that immediately after P9 stimulation, timing of PI1 and PI2 bursts tended to precede the timing of the P1N and PPCN bursts, suggesting that PI1 and PI2 play critical roles in initiating the locomotor rhythm.

We only identified one member of the second class of interneurons: PI3 (soma size: ~27 μm, *n* = 3; Fig. 5Q to U). The axon of PI3 projected to the contralateral pedal ganglion through the pedal commissure (Fig. 5Q). PI3 received cyclic excitation and inhibition during locomotor programs elicited by P9 stimulation, and became rhythmically active during the latter part of the locomotor program (Fig. 5R). Specifically, it fired at 342° ± 3.2° (*n* = 3), which is approximately the time phase II P1Ns are active. Stimulation of PI3 for prolonged periods enhanced locomotor programs (Fig. 5S and T, *n* = 3). Stimulation of PI3 also elicited fast excitatory postsynaptic potentials (EPSPs) in contralateral P1Ns that were mediated by a combination of chemical and electrical transmission (Fig. 5U). Interestingly, those P1Ns that were postsynaptic to PI3 fired at phases around 345.7° ± 27° (*n* = 3), which are near the cluster center of phase II.

**Fig. 7.**
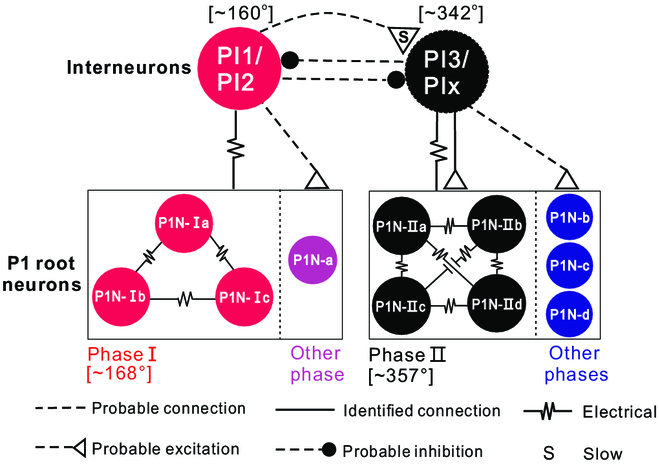
A schematic diagram showing the postulated central pattern generator (CPG) for *Aplysia* locomotor rolling wave. We suggest that the rolling wave is generated by a half-center oscillator composed of interneurons PI1/PI2 that fire near 160°, and interneurons PI3/PIx that fire near 342°. PIx is a hypothesized interneuron that would fire with PI3 and throughout the locomotor program. We suggest that the transition from PI1/PI2 to PI3/PIx activity is accomplished by slow excitation from PI1/PI2 to PI3/PIx (as illustrated) and/or other mechanisms, e.g., post-inhibitory rebound and adaptation (not illustrated) (see Discussion). PI1/PI2 and PI3/PIx are electrically coupled to coactive P1Ns, i.e., P1N-Is or P1N-IIs, respectively. PI3 also elicits a chemical EPSP on P1N-IIs. We postulate that neurons that fire at other times receive chemical excitation from PI1/PI2 or PI3/PIx. For simplicity, dorsal pedal neurons are not illustrated. Solid lines are synaptic connections described in the current work. Dashed lines are postulated connections. See Discussion for details.

The 3 interneurons, PI1 to PI3, represent the first identification of any interneurons in the *Aplysia* pedal ganglion. With a postulated PIx that is activated in the same phase as PI3, but throughout programs, we propose that a locomotor central pattern generator (CPG) based on a half-center oscillator combined with electrical coupling between P1Ns and between interneurons and P1Ns generates different phasic motoneuronal activity for the pedal wave in *Aplysia* (Fig. 7, see Discussion for detailed justification).

## Discussion

In this report, we used in vivo and in vitro electrophysiological and computational techniques to elucidate neural mechanisms underlying the generation of the rolling wave in *Aplysia*. In particular, we describe a cluster of newly identified pedal neurons that generate the rolling wave and 3 newly identified interneurons that contribute to rhythm generation and the creation of the phase shift. The locomotor programs we studied include those occurring spontaneously and those elicited by NaCl in intact animals or by tail nerve (P9) stimulation in the isolated CNS. These locomotor programs could correspond to 2 forms of locomotion: crawl or gallop, respectively, as distinguished in previous studies [26–28].

### Motor organization and the generation of the pedal wave in *Aplysia*

Rolling waves are the most common form of locomotion for axial locomotion. Examples include pedal waves in invertebrates (present in some species of gastropod molluscs and insect larvae), and body waves that are more common, and are present in both invertebrates (e.g., the leech) and vertebrates (e.g., lamprey and fish) [4–11]. These forms of axial locomotion differ in that different body parts are used. In some species, it is the foot; in other species, it is the body. Additionally, the direction of the wave varies. For example, the pedal wave in *Aplysia* is a retrograde wave in that muscle contractions are initiated anteriorly and then progress toward the tail of the animal, i.e., they move in a direction that is the opposite of the direction of movement [4]. In contrast, the pedal wave in *Drosophila* larvae is an anterograde wave in that muscle contractions and movement are in the same direction [9]. Despite these differences, rolling waves are all similar in that they are characterized by rhythmic contractions of different body parts or segments, and there are phase shifts between bursts of activity.

In this study, we determined the phase of a peripheral nerve (PPCN/P10) in intact animals, identified a cluster of pedal motoneurons (P1Ns), characterized their activity during locomotor programs, and determined synaptic interactions that are responsible for the pedal wave in *Aplysia*. Although previous investigators [22] recorded from the PPCN/P10 in intact animals, these authors did not formally relate bursts of activity in PPCN/P10 to muscle contractions. We now show that PPCN activity occurs during tail/posterior foot contraction (Fig. 1A to J). Previous work extensively probed neurons on the dorsal surface of the pedal ganglion [4,17,18,21,29,30] and reported that their activity covers a complete cycle of the locomotor rhythm [18]. We obtained similar results and further showed that these neurons are difficult to characterize as identified neurons since they are broadly distributed and intermingled with nonrhythmic neurons (Fig. 2E). Consequently, it is difficult to address basic questions such as how many of these neurons are motoneurons.

In contrast, we identified a cluster of 20 to 30 P1Ns on the ventral surface of the pedal ganglion that are more amenable to further investigation. Activity in P1Ns represents the locomotor rhythm, and their activity is clustered around 2 phases: phase I (around 168.49°) and phase II (around 357.13°) (Table and Fig. 2J). Similar results were obtained for dorsal pedal neurons except that, on the dorsal surface, the peak near phase I was larger, i.e., it was large enough so that the data were also consistent for a single peak cluster (Table and Fig. 2D). Overall, our data indicate that dorsal and ventral neurons could be controlled by the same (or a similar) pattern-generating mechanism (see the next section).

Because P1Ns are re-identifiable, we were able to further characterize them. Importantly, we developed a neuromuscular preparation and demonstrated that the majority of these cells are motoneurons (Fig. 3). Additionally, P1Ns that are coactive tend to be electrically coupled (Fig. 4). These results provide a basis for elucidating pattern-generating mechanisms for the rolling wave.

### Rhythm- and pattern-generating mechanisms for the pedal rolling wave in a nonsegmental CNS

In addition to identifying locomotor motoneurons, we also characterized 3 interneurons on the ventral surface of the pedal ganglion. These are the first pedal wave interneurons that have been characterized in *Aplysia*. In considering how pedal waves are generated in general, there are 2 questions that are of particular interest: (a) how is the rhythm generated, and (b) what produces the phase shift between movements of different body parts, or segments. In animals with a segmented CNS, with the possible exception of the small nervous system of *C. elegans* that does not have CPG interneurons [10], each CNS segment typically contains an independent CPG that generates its own rhythm. Phase shifts between segments are accomplished by differential coupling, i.e., gradient or asymmetrical excitatory projections between segmental CPGs [31–36]. The underlying circuit and synaptic basis for asymmetrical excitatory projections has been most extensively characterized in the crayfish swimmeret system [37–39]. This is, however, a type of appendicular, rather than axial, locomotion.

In molluscs, such as *Aplysia*, locomotor behavior is mediated by a single nonsegmented pedal ganglion. Our data support the idea that behavior is mediated by a single CPG based on a half-center oscillator. First, Rayleigh tests and cluster analyses of the phasing of populations of both dorsal and ventral pedal neurons demonstrated that the activity of these neurons is clustered so that 2 phases of a motor program are created (phases I and II) (Table and Fig. 2D and J). Second, the 2 cluster centers of phases I and II matched the activity phasing of 3 individual interneurons, i.e., PI1 and PI2 were active during phase I, and PI3 was active during phase II (Fig. 5). Thus, the data support the idea that the activity of pedal neurons is controlled, at least in part, by a pattern-generating network formed by the 2 classes of PIs. Indeed, there is extensive electrical coupling between PI1/PI2 and P1Ns that are active during phase I (Fig. 5A to P), and between coactive P1Ns (Fig. 4). The synapse from PI3 to P1Ns (active near phase II) is mixed, with both a chemical and an electrical component (Fig. 5Q to U), suggesting that chemical synapses also play roles in controlling motoneuronal activity. These patterns of synaptic connections could contribute to the phasing of motoneuronal activity. More importantly, inactivation of PI1 (Fig. 5B and E) or activation of PI2 (Fig. 5N) and PI3 (Fig. 5S) can reduce or increase the cycle frequency of locomotor programs, respectively, supporting a role for these PIs in locomotor rhythm generation.

Consequently, we propose that the half-center oscillator is composed of PI1/PI2 and PI3. During the initial cycle of a locomotor pedal wave following P9 stimulation, PI1/PI2 activity is likely initiated first because PI1/PI2 activity precedes activation of the P1Ns and activity in the PPCN (Fig. 6). Moreover, PI1/PI2 (Fig. 5G, H, L, and M) receive out-of-phase inhibition, as would be the case if they were part of a half-center oscillator. Because PI3 is only rhythmically active during a portion of the locomotor program (Fig. 5Q to U), it is likely that additional, as yet unidentified, interneurons fire during phase II. We refer to this type of cell as PIx and suggest that it would provide the primary out-of-phase inhibition of PI1 (Fig. 5G and H) and PI2 (Fig. 5L and M). Thus, PI1/PI2 and PI3/PIx would mutually inhibit each other (Fig. 7). We currently do not have data that indicate how phase transitions would occur, but we suggest that they would be mediated by either slow excitation from PI1/PI2 to PI3/PIx (Fig. 7) or the presence of post-inhibitory rebound and adaptation in the circuit as demonstrated in other half-center networks [40–46]. Thus, this CPG model would explain how the rhythm of the *Aplysia* pedal wave is generated.

In terms of pattern generation, i.e., how specific phases of different motoneurons or P1Ns are mediated, our data suggest that interneurons primarily drive coactive motoneurons via relatively strong electrical coupling (Fig. 7). Electrical or chemical connections are probably weaker between interneurons and P1Ns that are activated with a delay (e.g., Fig. 5U). Electrical coupling between populations of either phase I or II PINs also contributes (Fig. 4). Thus, this model provides a parsimonious account for both rhythm generation and phase shifts between P1Ns and highlights the importance of electrical coupling. Notably, electrical coupling between motoneurons, and between motoneurons and interneurons, has been described in a number of model systems [47], including vertebrate systems [48], and may contribute to pattern generation. In addition, computational analysis of population activity of dorsal pedal neurons [30] suggests the presence of a spiral attractor network in the pedal ganglion. Our identification of a single CPG in the pedal ganglion could be a physical implementation of this attractor.

Another feature of the network that could also contribute to the phase shift between pedal neurons is the complexity of the PI1/PI2 axon, which makes a loop within the ipsilateral pedal ganglion. Conceivably, the neuropilar postsynaptic sites of pedal neurons active during different phases could be arranged so that they are activated sequentially, possibly more so for dorsal neurons that have less electrical coupling (see [18,30]). PI1/PI2 differ from most other PIs that have been identified in gastropod molluscs (*Clione*, *Melibe*, and *Dendronotus*) [40,41,49–52]. Most commonly, PIs have projections that exit the pedal ganglion and travel to either the contralateral pedal ganglion or the cerebral ganglion. In contrast, the PI1/PI2 axons remain in the ipsilateral ganglion. Given that PI1/PI2 appear to play roles in generating motor programs, axon paths of PI1/PI2 are consistent with previous work showing that a single pedal ganglion is sufficient to generate locomotor rhythmic activity [21]. On the other hand, PI3’s contralateral projection suggests that it could play a role in coordinating rhythmic activity between left and right pedal ganglia.

Future work is needed to make the network model complete. At present, we have been unable to locate some interneurons, particularly one member of class 2 interneuron, i.e., PIx, perhaps because the cell body of PIx is not located on the surface of the pedal ganglion. This is one direction the future work shall focus on. It will be important to identify PIx and characterize its synaptic connections underlying rhythm generation of the CPG.

It is worthwhile to compare the *Aplysia* locomotor network to locomotor networks in other gastropod molluscs [53,54]. In addition to muscular crawling (i.e., the pedal wave in benthic *Aplysia*), some other benthic gastropods crawl through mucociliary locomotion. This occurs in *Tritonia* [55] and *Pleurobranchaea* [56]. In contrast, pelagic gastropods such as *Clione* locomote through wing flapping [57,58]. Both mucociliary locomotion and wing flapping are classified as appendicular locomotion. Other benthic gastropods occasionally swim through either left-right (*Melibe* and *Dendronotus*) [51,52] or dorsal-ventral body flexions (*Tritonia* and *Pleurobranchaea*) [59–61]. These forms of locomotion are classified as axial. Given that gastropod mucociliary locomotion is presumably nonrhythmic, it will not be discussed further. Although the other forms of locomotion are not rolling waves, they are all rhythmic and, in some cases, are mediated by single CPGs in the pedal ganglion. This is true for *Clione* [40,41,49,50] and *Dendronotus* [52]. In *Melibe*, the behavior is mediated by both the cerebral and pedal ganglia [51,52]. In *Tritonia* and *Pleurobranchaea*, it is exclusively mediated by the cerebropleural ganglion [61–65]. Despite diversity in the behavior and mediating ganglia, all of these CPGs include electrical coupling and consist of single half-center oscillators that generate phasic activity [53,54].

In summary, our findings suggest that *Aplysia* locomotor behavior is mediated via activation of a half-center oscillator that is responsible for both rhythm generation and creating the phase shift that is important for the rolling wave*.* This is the first demonstration of this type of organization in a CNS that is not segmented. In species that have a segmented CNS, phase shifts are mediated by gradient or asymmetrical excitatory projections between coupled CPGs. Our results indicate that electrical coupling among motoneurons and between interneurons and motoneurons plays a critical role in generating phase shifts within a single CPG. It is possible that synaptic sites of circuit elements within a single ganglion are relatively close and enable electrical coupling to become an effective mechanism for rolling waves. Thus, our study opens a line of research, in this and other model systems, to determine more extensively how electrical and chemical synapses may play various roles in mediating rolling waves in animals with either a segmented or nonsegmented CNS.

## Materials and Methods

### Subjects and electrophysiology

Experiments were performed on *Aplysia californica* (100 to 300 g) obtained from Marinus (Newport Beach, CA, USA). *Aplysia* are hermaphroditic (i.e., each animal has reproductive organs normally associated with both male and female sexes). Animals were maintained in circulating artificial seawater (ASW) at 14 to 16 °C with a 12 h day-12 h night cycle.

Intracellular recordings were made using single-barrel electrodes (5 to 10 MΩ) filled with 0.6 M K_2_SO_4_ and 60 mM KCl. Intracellular signals were acquired using an AxoClamp 2B or 900A amplifier (Molecular Devices), a Neuroprobe amplifier (model 1600; A-M Systems), or a Getting model 5A amplifier. A Grass model S88 stimulator was used for stimulation. Extracellular signals were acquired from polyethylene suction electrodes using a differential alternating current amplifier (model 1700; A-M Systems). Recordings were made in ASW (460 mM NaCl, 10 mM KCl, 55 mM MgCl_2_, 11 mM CaCl_2_, and 10 mM HEPES buffer, pH 7.6) unless otherwise indicated. All chemicals were purchased from Sigma (St. Louis, MO).

### Implanted electrodes in intact animals

In order to record activity from the PPCN in intact animals, we implanted a cuff electrode [66], which was made of polyethylene tubing (PE100), with one end heat-pulled to create a small tip that fit over the PPCN. Animals were anesthetized by injecting 333 mM MgCl_2_ at 50% of their body weight. Prior to injection, MgCl_2_ was cooled to 4 °C. After they were injected, animals were placed in a pan with iced ASW and a 2-cm cut was made on the side of body near the foot. The PPCN was located from the dorsal side and was cut. The proximal end of the nerve was then suctioned to the tip of the cuff electrode with a syringe, and the open end of the cuff electrode was cut short (i.e., cut to about 1 cm in length). The bare tip (2 to 3 mm) of a stainless-steel wire electrode (A-M Systems, 0.002" bare, 0.0045" Teflon coated) was inserted into the open end of the cuff electrode. Then, both ends of the cuff electrode were sealed with a cyanoacrylate glue, and the electrode was glued to the body wall muscle with the same glue. Note that cyanoacrylate glue was used in these experiments because this glue was normally used to repair coral reef in fish tanks, and is appropriate for soft tissues involving seawater. A second stainless-steel electrode with a bare tip was also glued to the body muscle and used as the reference electrode. After surgery, the cut skin was sutured and then glued together to prevent leakage of hemolymph. Animals were returned to their home tank, and usually recovered and resumed normal activity, including locomotion, within 24 to 48 h.

During recording sessions, *Aplysia* were placed in a clear tank. Part of the tank was wrapped with aluminum foil, which was used as a noise reducing shield. The movement of the foot and the body was videoed from the bottom of the tank with a Canon EOS 650D camera. PPCN activity was recorded at the same time with an A-D converter (Axon) after amplification with the Differential AC 1700 Amplifier (A-M Systems). We recorded spontaneous locomotion and defensive locomotion elicited by applying NaCl crystals to the tail. Locomotor movement was quantified by computing the position of the posterior foot/tail or the front foot in a frame every 2 s. For example, to measure the position in pixels, we used software that indicated the *x* and *y* positions of the pixel in each frame for either the posterior foot (indicated by a red “x” in Fig. S1A) or the front foot (indicated by a red dot in Fig. S1A) at different time points, i.e., *x*_i_, *y*_i_ (0 s); *x*_i+1_, *y*_i+1_ (2 s); *x*_i+2_, *y*_i+2_ (4 s), etc. The pixel displacement values were calculated by the following formula: Sqrt ((*x*_i+1_ − *x*_i_)^2^ + (*y*_i+1_ − *y*_i_)^2^). These values were then converted from the displacement in pixels to displacement in centimeters based on the scale bar.

### Nerve backfills

Backfills were performed as described previously [67]. Briefly, the cut end of the PPCN was backfilled 1 to 2 days at 15 °C with 5% biocytin in a small well made with silicone grease (Molycote). After a wash, ganglia were fixed in 4% paraformaldehyde and desheathed to expose cell bodies. The ganglia were then processed with fluorescein isothiocyanate (FITC)-avidin for 1 to 2 days to develop the backfilled neurons. A fluorescence microscope (Nikon or Olympus) was used to view and photograph the ganglion.

### Cell identification

P1Ns and PIs, PI1, PI2, and PI3, are all on the ventral surface of the pedal ganglion and are described here for the first time. To reveal the morphology of these newly identified neurons, we iontophoretically filled them with either 3% 5(6)-carboxyfluorescein dye in 0.1 M potassium citrate, Alexa Fluor 488 (green), or Alexa Fluor 568 (red). Currents used to inject the carboxyfluorescein ranged from −5 to −8 nA, and injections lasted 10 to 20 min. The Alexa dyes were injected using −7 nA for 10 min. A fluorescence microscope (Nikon or Olympus) was used to view and photograph ganglia.

### Neuromuscular preparations in semi-intact animals

Animals were anesthetized by injecting 333 mM MgCl_2_ (~50% of body weight). Pedal ganglia were removed maintaining the innervation of the foot muscles. This innervation extends from the posterior edge of the mantle cavity to the anterior tentacles and parts of the body wall. Preparations were pinned to a 2-chamber dish lined with Sylgard (Dow Corning) (Fig. 3A). To facilitate the pinning, we only preserved the innervation of the experimental (mostly right) side of the preparation. The pedal nerves on the other side were severed. The foot muscle was situated in the larger of the 2 chambers, whereas the pedal ganglion was pinned to the smaller chamber, which had a higher Sylgard floor.

We cannulated the pedal artery, and foot muscles were continuously perfused with fresh ASW at ~0.5 ml/min throughout the experiments. Perfusion began immediately after the preparation was isolated. Experiments were initiated after 2 to 3 h (i.e., washout of the anesthetic). To monitor muscle contractions elicited by either P9 stimulation or motoneuronal stimulation, the edge of foot was pinned with multiple pins, and the middle of the foot was attached to a force transducer (Isotonic Transducer “60–3000,” Harvard Apparatus) (see Fig. 3A). Preparations were maintained at 14 to 16 °C.

### Isolated CNS preparations and motor programs

Electrophysiological recordings from CNS preparations (the cerebral and pedal ganglia in some cases, or the pedal ganglia only in others) were performed as described previously [20,68–73]. Animals were anesthetized by injection of 333 mM isotonic MgCl_2_ (~50% of body volume), and the cerebral and pedal ganglia were dissected out. Ganglia were desheathed, transferred to a recording chamber (lined with Sylgard) containing ~1.5 ml of ASW, continuously perfused at 0.3 ml/min, and maintained at 14 to 17 °C. To suppress polysynaptic pathways, a high divalent cation saline (HiDi) was used containing the following: 368 mM NaCl, 10 mM KCl, 13.8 mM CaCl_2_, 101 mM MgCl_2_, and 10 mM HEPES, and pH at 7.6. This saline does not alter postsynaptic potential amplitude [74]. To block chemical synaptic connections, we used a 0 Ca^2+^ saline containing the following: 368 mM NaCl, 10 mM KCl, 101 mM MgCl_2_, and 10 mM HEPES, and pH at 7.6. This solution had no obvious effects on electrical connections. In experiments without Ca^2+^, we made sure that the membrane potential throughout experiments remained the same by applying appropriate hyperpolarizing or depolarizing currents. The cell hyperpolarization/depolarization was performed using the single electrode current-clamp technique, and care was taken to correctly balance the electrode resistance.

Locomotor programs in the isolated CNS were elicited by stimulation of the tail nerve (P9) at 10 Hz using pulses that were 10 V and had a duration of 10 ms. The stimulation lasted a total of 1 to 2 s.

### Data and statistical analyses

Electrophysiological recordings were digitized online with Axoscope (Molecular Devices) and plotted with CorelDraw (Corel). Because the PPCN contains axons from 3 or more neurons, its bursting frequency was analyzed in 3-s bins. For activity in PPCN and pedal neurons, mean frequency is the total number of spikes divided by the duration of a specific time period. The time period was calculated after nerve stimulation for evoked programs or over the entire duration for spontaneous programs. Intraburst frequency was calculated by determining the number of spikes during a burst divided by the burst duration. If there was more than one burst, intraburst frequency was averaged. Note that mean values of PPCN frequency and posterior or front foot displacement over the entire recording illustrated, which were plotted as red horizontal lines in Fig. 1D to F, O, and P and Fig. S1, are used to mark the beginning and the end of PPCN bursts or foot displacement. Coupling ratios for electrical coupling were defined as the voltage change of a postsynaptic neuron divided by the voltage change of a presynaptic neuron, i.e., the one receiving the current injection. A pair of neurons was operationally defined as not electrically coupled when their coupling ratio was below 0.002.

Bar graphs and scatter diagrams were plotted using Prism software (GraphPad). Data are expressed as the mean ± SEM. All experimental data were taken from individual animals or preparations, and *n* refers to the number of preparations unless otherwise stated. Statistical tests were performed as appropriate using Prism software. Tests used include Student’s *t* tests and one-way analyses of variance (ANOVAs). Data that showed significant effects in ANOVAs were further analyzed by making individual comparisons using a Bonferroni’s correction.

### Statistics and cluster analysis for circular data

To test uniformity for circular data, we considered a data sample of size *n* containing values {*α*_1_, *α*_2_, …, *α*_n_} in the range [0, 2*π*) (equivalent to phases of [0°, 360°) as used in the other sections of the paper). Specifically, an important issue we addressed was whether the phase distribution of pedal neurons over one cycle [0°, 360°) is uniform. We used the Pycke test for this purpose [75]. For the Pycke test, the test statistic *V* [76] was given by:

$$V=\frac{1}{n}\overset{n}{\underset{i=2}{\Sigma }}\overset{i}{\underset{j=1}{\Sigma }}\frac{2\left(\cos\left(a_{i}-a_{j}\right)-\sqrt{0.5}\right)}{1.5-\left(2\sqrt{0.5}\cos\left(a_{i}-a_{j}\right)\right)}$$

Then, the *P* value of each test was calculated using simulation as described previously [75]. We drew *M* = 999 sets of pseudo-samples of size *n* from a uniform distribution on [0, 2*π*). For each set of pseudo-samples, we calculated the test statistic, *V*. The total number of pseudo-samples with their V equal to or greater than that of the real sample was counted and represented as *Q*. The *P* value of each test was then given by (*Q* + 1)/(*M* + 1). For *P* < 0.05, the circular data are nonuniform.

The Rayleigh test [77] is widely used to determine whether this is a single cluster in data samples [78]. It was run using the *rayleigh.test* function in *R*. If *P* < 0.05, there is one peak in the circular data. In specific cases, the Rayleigh test is also useful for determining whether there is more than one cluster in circular data [77]. Specifically, if there are multiple symmetrical clusters in the data sample (e.g., 2 clusters that are 180° apart, or 3 clusters that are 120° apart), data can be tested after the appropriate transformation (e.g., a 2-fold transformation to test for 2 symmetrical clusters, and a 3-fold transformation to test for 3 symmetrical clusters [77]). To be more specific, to test for 2 symmetrical clusters that are 180° apart, the original phase data are multiplied by a factor of 2, and thus, the 2 original clusters are now 360° apart and actually overlap on a circle. The transformed data are tested with Rayleigh test, and if *P* < 0.05, there are 2 symmetric peaks in the original data.

For circular data with one cluster, their center could be directly determined by calculating the angle of the Rayleigh vector over the sample data ($\sum_{i}^{n}e^{j\varphi_{i}}/n$, where *φ*_i_ was sample datum and *j* is the imaginary unit) [78,79]. For the 2 symmetric clusters of 180° apart, after 2-fold transformation, there was now one cluster in the data sample, and the center of the transformed data sample could be determined directly by calculating the angle of the Rayleigh vector, *φ*_2-fold,center_; the original 2 centers must then be *φ*_2-fold,center_/2 and *φ*_2-fold,center_/2 + 180°.

Since Rayleigh tests run on transformed data only apply to data with 2 or more symmetrical clusters, we used another independent method that does not require 2 or more cluster centers to be symmetrical. Specifically, we adopted the *K*-means clustering to determine the phase/peak/cluster centers: (a) For each data sample of *n* size, given the number of clusters *k*, we randomly selected *k* points as the initial values of *k* cluster centers (*φ*_k,center_); (b) each point in the sample was attributed to the cluster with the least angular distance; (c) for points in each cluster (*φ*_k,i_), a new *φ*_k,center_ was determined by the angle of the Rayleigh vector, $\sum_{i}^{n_{k}}e^{j\varphi_{k,i}}/n_{k}$, with *j* the imaginary unit; (d) steps (b) and (c) were repeated until *φ*_k,center_ no longer changed. At this time, the clustering was finished and each point was assigned to different clusters. As the clustering results sometimes might be affected by the selection of the initial *k* points, we performed 10 independent clustering given *k* = 2. We found that during the 10 independent clustering trials when there were 2 clusters, some data points between clusters (shown in gray vertical lines in phase distribution histograms; see Figs. 2D and J and 4B) might be assigned to different clusters on different trials. At the conclusion of these 10 trials, these data points were excluded, and *φ*_k,center_ for each cluster was recalculated with data points that remained unchanged during 10 independent clustering trials, and this result was shown at the top of histograms (see Figs. 2D and J and 4B). To determine robustness of *K*-means clustering for circular data, we performed more extensive analysis with *K*-means clustering on these physiological data (Supplementary Materials, Table S1, and Fig. S3) and evenly or randomly distributed artificial data with the same sizes as the physiological data (Table S1 and Fig. S4).

The computation for Pycke test and *K*-means clustering was implemented with Python, while the Rayleigh tests after 2- and 3-fold transformation were implemented with the* rayleigh.test *function in *R*. The computer codes will be provided upon reasonable request.

## Data Availability

All data needed to evaluate the conclusions in the paper are present in the paper and/or the Supplementary Materials. The custom computer codes (Python) used for analyses for the circular data are available upon request (T.W., tao.wang@nju.edu.cn). Additional data related to this paper may be requested from the authors.

## Supplementary Materials

Supplementary 1 Fig. S1. Relationship between the onset of PPCN bursts and the onset of the front foot/posterior foot (tail) displacement. Fig. S2. A composite image of a backfill of the PPCN from 3 individual fluorescent images of the dorsal surface showing the location of filled somata. Fig. S3. Results for actual data from Figs. 2D and J and 4B partitioned into 2 or 3 clusters. Fig. S4. Results for evenly and randomly distributed artificial data with the same sample size as Fig. 2D partitioned into 2 or 3 clusters. Table S1. Summary data of the excluding rate for *K*-means clustering simulations performed (see Figs. S3 and S4). Text S1. The robustness of *K*-means clustering for circular data.

Supplementary 2 Movie S1. A video of an *Aplysia*’s locomotion following NaCl application (related to Fig. 1 and Fig. S1).
