# Supplementary material for: A Single Central Pattern Generator for the Control of a Locomotor Rolling Wave in Mollusc Aplysia
Source: Research (Wash D C). 2023 Mar 6;6:0060. doi: 10.34133/research.0060 (PMC10013812; doi:10.34133/research.0060)
Supplement: Supplementary 1 — Fig. S1. Relationship between the onset of PPCN bursts and the onset of the front foot/posterior foot (tail) displacement. Fig. S2. A composite image of a backfill of the PPCN from 3 individual fluorescent images of the dorsal surface showing the location of filled somata. Fig. S3. Results for actual data from Figs. 2D and J and 4B partitioned into 2 or 3 clusters. Fig. S4. Results for evenly and randomly distributed artificial data with the same sample size as Fig. 2D partitioned into 2 or 3 clusters. Table S1. Summary data of the excluding rate for K-means clustering simulations performed (see Figs. S3 and S4). Text S1. The robustness of K-means clustering for circular data. [file research.0060.f1.docx]

Supplementary Materials for

**A Single Central Pattern Generator for the Control of a Locomotor Rolling Wave in Mollusc *Aplysia***

Hui-Ying Wang, Ke Yu, Zhe Yang, Guo Zhang, Shi-Qi Guo, Tao Wang, Dan-Dan Liu, Ruo-Nan Jia, Yu-Tong Zheng, Yan-Nan Su, Yi Lou, Klaudiusz R. Weiss, Hai-Bo Zhou*, Feng Liu*, Elizabeth C. Cropper, Quan Yu*, Jian Jing*

* Corresponding authors. Email: jingj01@live.com (J.J.); yuq@pcl.ac.cn (Q.Y.); fliu@nju.edu.cn (F.L.); haibozhou@nju.edu.cn (H.B.Z.)

**This file includes:**

Supporting text

Figs. S1 to S4

Table S1

Legend for Supplementary video 1

**Other supporting materials for this manuscript include the following:**

Supplementary video 1

Supplementary Materials

**Fig. S1.**


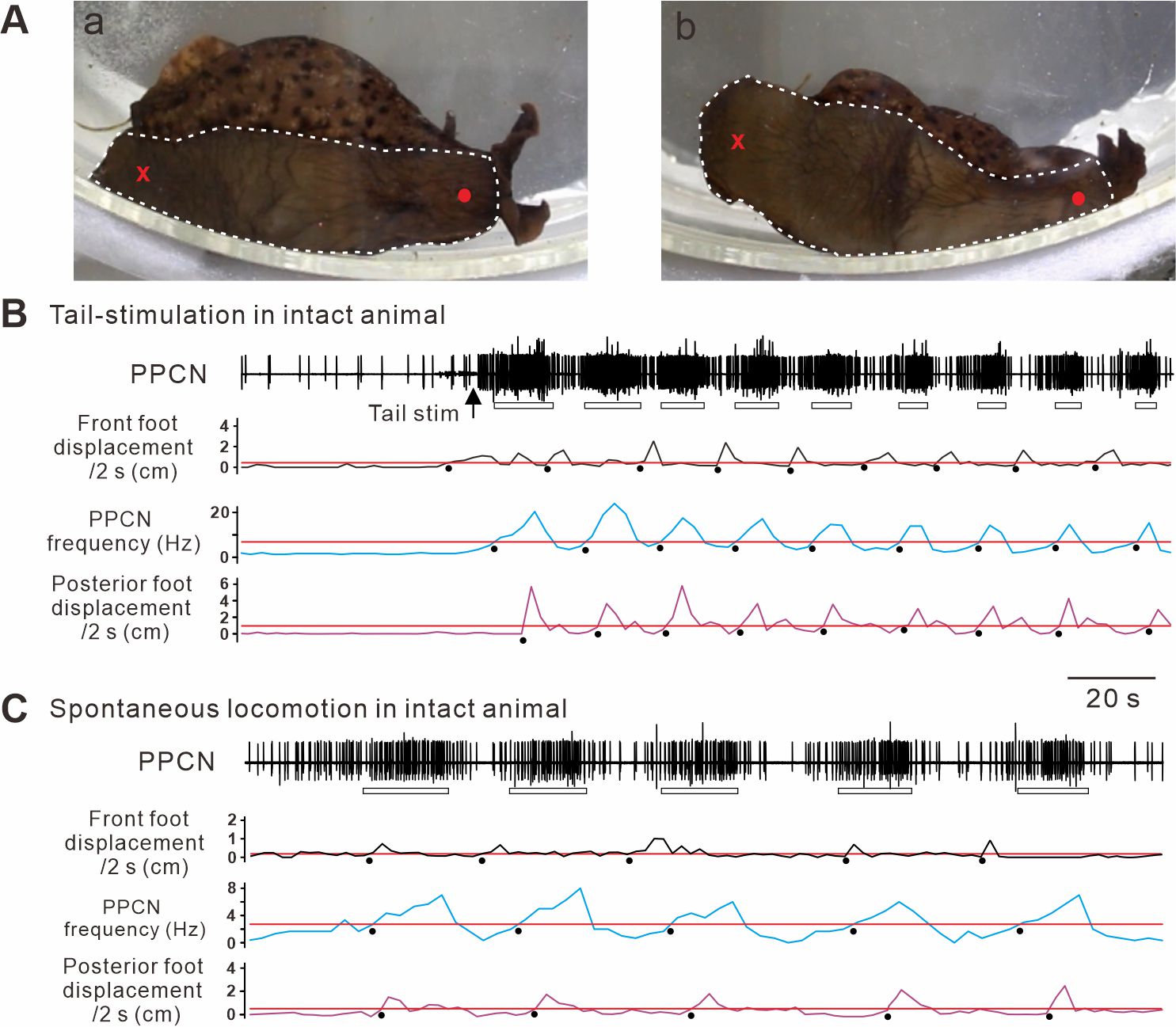


**Fig. S1.** Relationship between the onset of PPCN bursts and the onset of the front foot/posterior foot (tail) displacement. (A) Two images of an *Aplysia* from video frames showing the position of the posterior foot/tail (indicated by a red “x”) and the front foot (indicated by a red dot) used for measurement (see Methods). The *Aplysia* was videotaped from the bottom of the tank. White dashed lines outline the foot. (B and C) PPCN activity and movement data from intact animals during NaCl-triggered locomotion (B), and spontaneous locomotion (C). The red horizontal lines (B-C) indicate the average PPCN firing frequency and foot displacement. Onsets of PPCN bursts or movement episodes are indicated by black dots. The data show that PPCN bursts began before periods of posterior foot/tail displacement and after periods of front foot displacement.

**Fig. S2.**


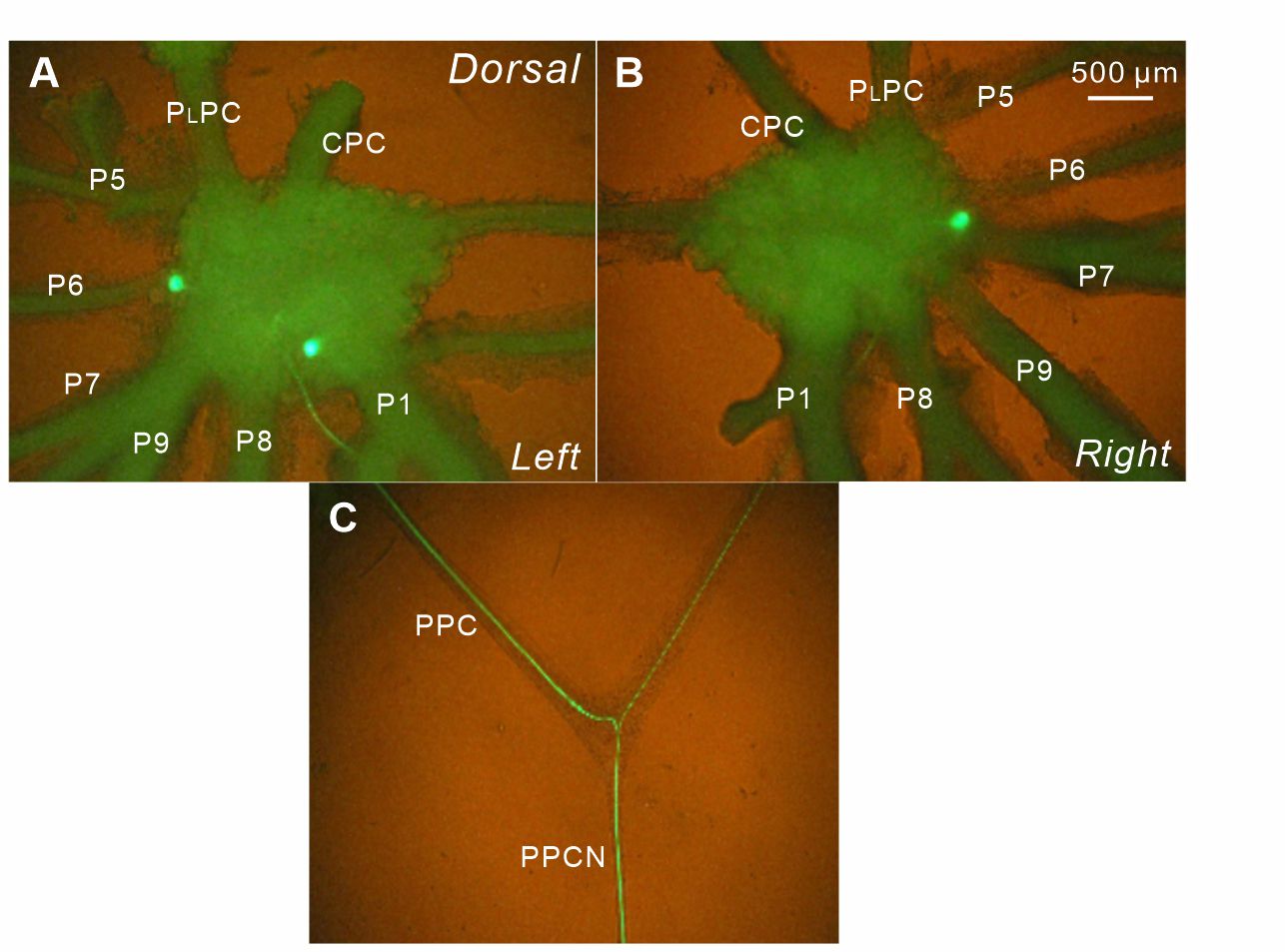


**Fig. S2.** A composite image of a backfill of the PPCN from three individual fluorescent images of the dorsal surface showing the location of filled somata. (A) The two cell bodies (green) were backfilled of the left pedal ganglion. (B) The one cell body (green) was backfilled of the right pedal ganglion. (C) Backfilled axons (green) in PPC and PPCN.

**Supplementary Movie**

**Supplementary video 1** (a separate file, Supplementary video 1.mp4). A video of an *Aplysia*’s locomotion following NaCl application. The animal was videotaped from the bottom of a clear, circular tank, with its foot, part of the head and right parapodium visible. A recording of PPCN/P10 activity from Axoscope digitizing software was overlaid at the bottom. Strong posterior foot/tail contractions were associated with strong PPCN/P10 bursting activity (see also Fig. 1C, D and Fig. S1B). The duration of the original video was 1 min 20 s. The current video was compressed to 80% with a duration of 1 min 04 s. NaCl crystals were applied on the tail at ~ 1 s. The right image in Fig. 1C corresponds to a frame near 42 s. (Related to Fig. 1 and Fig. S1)

**Supporting Information Text**

**The robustness of K-means clustering for circular data**

K-means clustering is a method of vector quantization that aims to partition n observations into k clusters (given k => 2) in which each observation belongs to the cluster with the nearest mean (cluster centers or cluster centroid), serving as a prototype of the cluster (Wikipedia). To determine the robustness of this method in partitioning our circular data for Fig. 2D, 2J, 4B, we performed simulations in 100 trials (instead of 10 trials in the main text), with the actual data, random-distributed data and evenly-distributed data of the same size, n (Table S1). We used the excluding rate to quantify the robustness of the K-means clustering. The excluding rate is the number of excluded sample data over the sample size, i.e., data samples that are not consistently assigned to a single cluster, but are assigned to one cluster during one trial, but to another cluster during another trial.

We note that during each simulation of the 100 trials, because K-means clustering could be sensitive to the initial assigning of clustering centers (which are generated with random numbers), occasionally for Fig. 2J actual data sample and Fig. 4B actual data sample in 2-cluster case (Fig. S3B and C), there are a few outlier trials that showed very different clustering than ones from other clustering results, i.e., the apparent cluster centers in these few trials are near the edge of the cluster, rather than near the center compared to most other clustering trials (Fig. S3E and F). Thus, when determining the excluding rate and the data samples that were assigned to different clusters during different trials, we only include sample data that were similarly classified into the different clusters over at least 5 trials. Outliers were not included because there are less than 5 outlier trials.

Randomly-distributed data were generated from a uniform distribution within [0, 2pi) using the function *numpy.random.uniform*() in Python. Evenly-distributed data were generated as a data set with an even spacing (or interval). Each data sample is the same size as the corresponding actual data sample. To evaluate the level of excluding rate for random-distributed data, we generated 1000 sets of data samples, calculated their resulting excluding rate for each data set (with 100 trials), and then provided average results of these 1000 data sets in Table S1.

Examples of analyses of circular data are shown for Fig. 2D (Two clusters: Fig. S3A, D, actual data; Fig. S4A, evenly-distributed data; Fig. S4B, random-distributed data. Three clusters: Fig. S3G, actual data; Fig. S4C, evenly-distributed data; Fig. S4D, random-distributed data). Fig. 2J (Two clusters: Fig. S3B, E, actual data. Three clusters: Fig. S3H, actual data), and Fig. 4B (Two clusters: Fig. S3C, F, actual data; Three clusters: Fig. S3I, actual data). The results (Table S1) indicate that the excluding rate was relatively low (< 0.2) when the actual data were partitioned into 2 clusters. For, all other data samples, the excluding rate was high (> 0.4). This supports the idea that the actual physiological data should be divided into 2 clusters.

**Table S1. Summary data of the excluding rate for K-means clustering simulations performed (see Fig. S3 and S4). Results for actual data are shown in blue.**

| Parameter | Data | *k* = 2 clusters | | | *k* = 3 clusters | | |
| --- | --- | --- | --- | --- | --- | --- | --- |
|  |  | Actual data | Even | Random (mean ± std of 1000 sets) | Actual data | Even | Random  (mean ± std of 1000 sets) |
| Excluding rate | Dorsal neurons (Fig. 2D, n = 71) | 0.0563 | 0.8732 | 0.5236 ± 0.2851 | 0.7887 | 0.9014 | 0.5955 ± 0.2603 |
|  | P1Ns  (Fig. 2J, n = 200) | 0.155 | 0.84 | 0.5481 ± 0.2889 | 0.47 | 0.88 | 0.5842 ± 0.2659 |
|  | Coupled PINs (Fig. 4B, n = 113) | 0.0177 | 0.8761 | 0.5214 ± 0.3003 | 0.5752 | 0.8673 | 0.5875 ± 0.2601 |

**Fig. S3.**


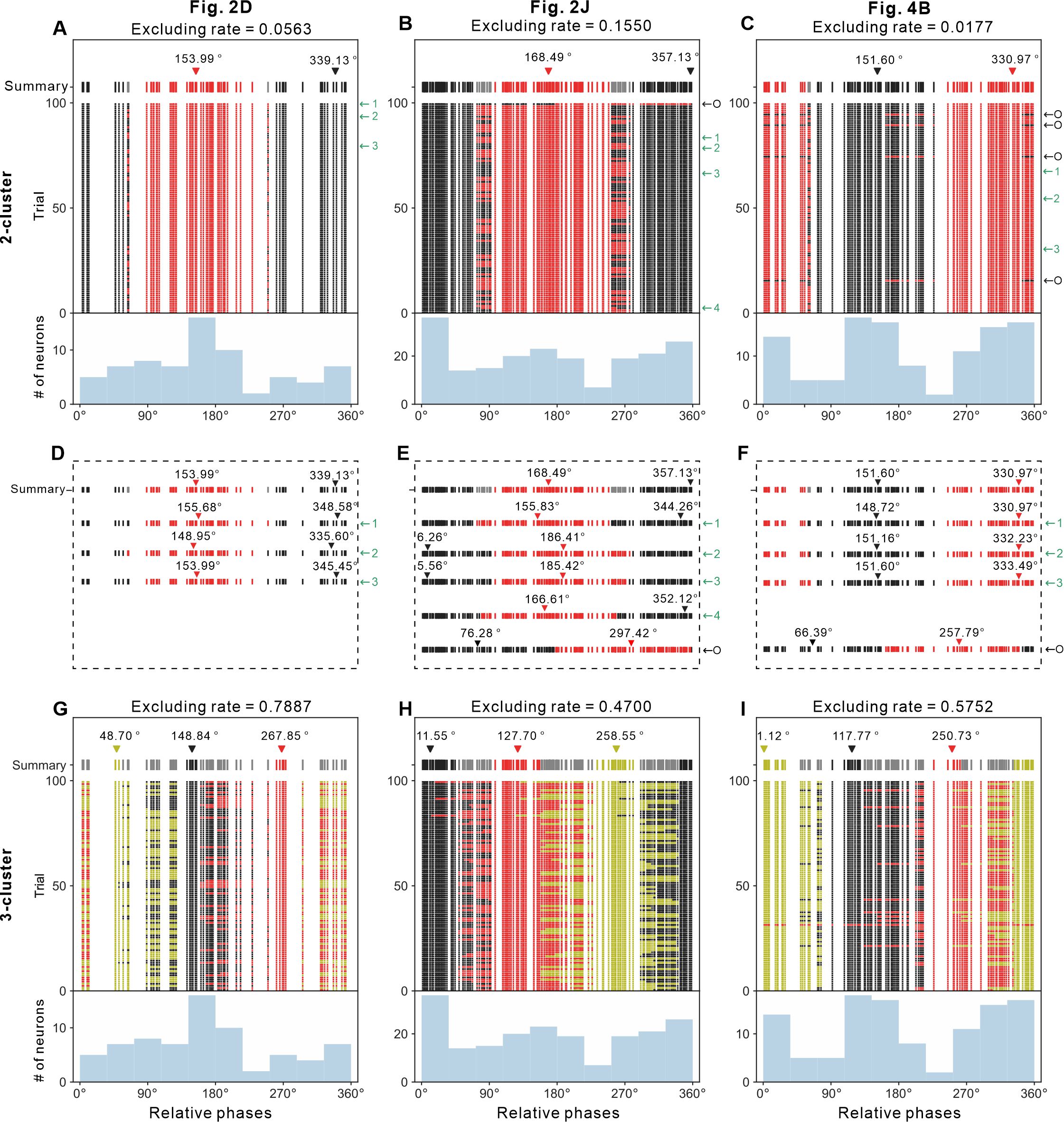


**Fig. S3.** Results for actual data from Fig. 2D, 2J, 4B partitioned into 2 or 3 clusters. Each panel except D to F is composed of the upper part (clustering results after 100 trials indicated by “Summary”, and the 100 clustering trials) and the lower part (a histogram of the data sample shown in blue). (A to C) Data partitioned into two clusters. Red and black bars denote members of the two clusters respectively. At the top (indicated by “Summary” to the left), the gray bars show excluded data which could not be stably assigned to a cluster. (D to F**)** The “Summary” and examples from individual trials as marked with green arrows and number in A-C. The phases at the top above the arrowheads denote cluster centers calculated for each trial. One of the outlier trials from (B and C) is illustrated at the bottom in (E and F), respectively. There is only one outlier trial in (B). There are four outlier trials in (C), all of which had the same cluster centers. Note that the cluster centers of the outlier trials are distinct from most other trials which had similar cluster centers. (G to I) Data partitioned into three clusters. Red, yellow and black bars denote members of the three clusters respectively. At the top (indicated by “Summary” to the left), the gray bars denote the excluded data which could not be stably assigned to a cluster. The phases at the top above the arrowheads indicate cluster centers. Outlier trials are indicated with black arrows and an “O” at the right in panels B, C, E and F. Note that the excluding rate is low when data are clustered into 2 (A to C), but is high when data are clustered into 3 (G to I).**Fig. S4**


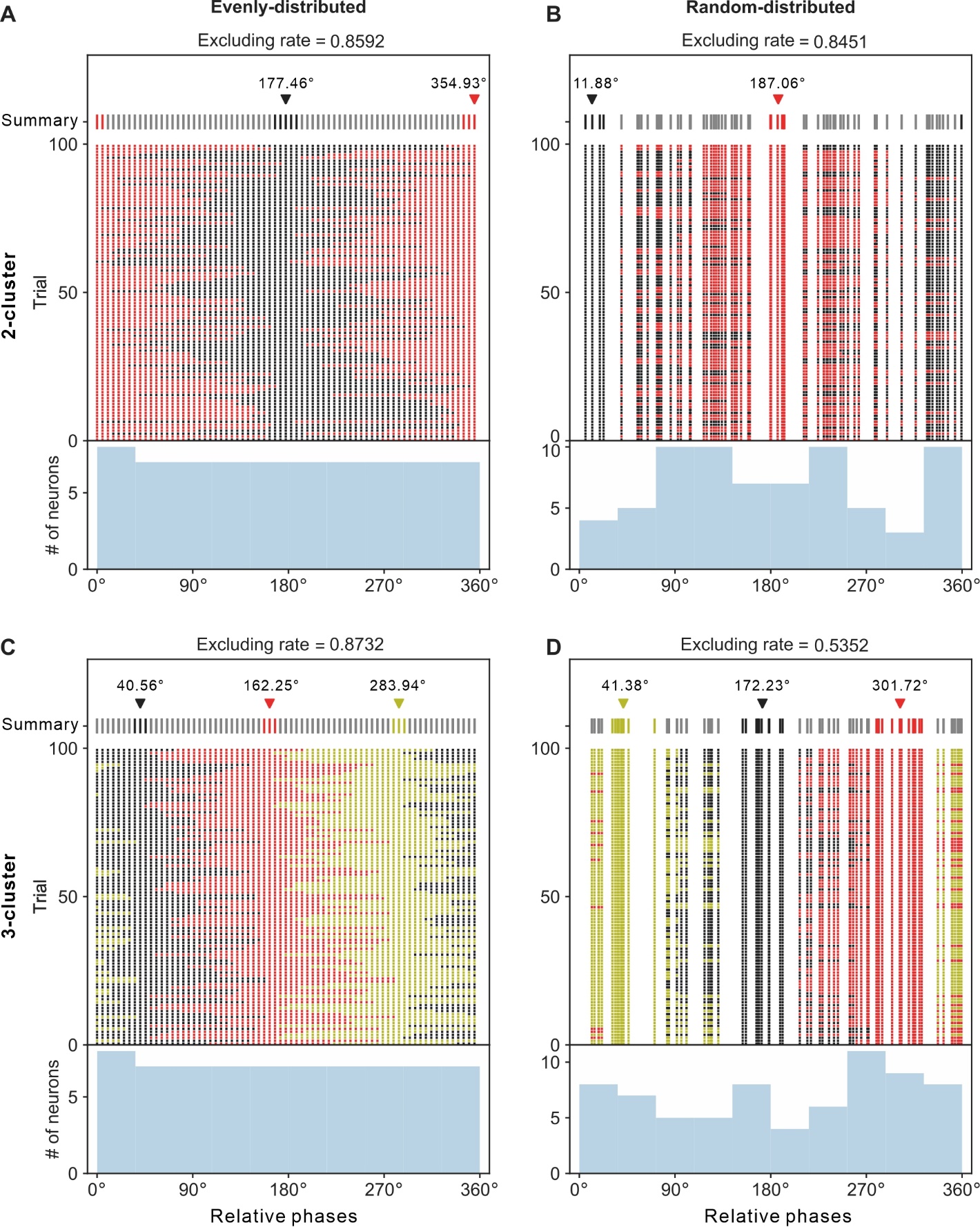


**Fig. S4.** Results for evenly- and random-distributed artificial data with the same sample size as Fig. 2D partitioned into 2 or 3 clusters. Each panel is composed of the upper part (clustering results after 100 trials indicated by “Summary”, and the 100 clustering trials) and the lower part (a histogram of the data sample shown in blue). (A and B) Data partitioned into two clusters. Red and black bars denote members of the two clusters respectively. At the top (indicated by “Summary” to the left), the gray bars denote the excluded data which could not be stably assigned to a cluster. (C and D) Data partitioned into three clusters. For random-distributed data (B and D), only results from one set among 1000 sets are illustrated. Red, yellow and black bars denote members of the three clusters respectively. At the top (indicated by “Summary” to the left), the gray bars denote the excluded data which could not be stably assigned to a cluster. The phases at the top above the arrowheads indicate cluster centers. Note that the excluding rate is high in all cases.
